# Maternal Dietary Patterns and Gestational Diabetes Mellitus in a Multi-Ethnic Asian Cohort: The GUSTO Study

**DOI:** 10.3390/nu8090574

**Published:** 2016-09-20

**Authors:** Jamie de Seymour, Airu Chia, Marjorelee Colega, Beatrix Jones, Elizabeth McKenzie, Cai Shirong, Keith Godfrey, Kenneth Kwek, Seang-Mei Saw, Cathryn Conlon, Yap-Seng Chong, Philip Baker, Mary F. F. Chong

**Affiliations:** 1 Liggins Institute, The University of Auckland, Auckland 1023, New Zealand; j.deseymour@auckland.ac.nz (J.d.S.); liz.mckenzie@auckland.ac.nz (E.M.); philip.baker@auckland.ac.nz (P.B.); 2 Department of Obstetrics & Gynaecology, Yong Loo Lin School of Medicine, National University of Singapore, Singapore 119228, Singapore; chiaairu@u.nus.edu (A.C.); obgcais@nus.edu.sg (C.S.); yap_seng_chong@sics.a-star.edu.sg (Y.-S.C.); 3 Singapore Institute for Clinical Sciences, Agency for Science, Technology and Research (A*STAR), Singapore 117609, Singapore; marjorelee_colega@sics.a-star.edu.sg; 4 Institute of Natural and Mathematical Sciences, Massey University, Auckland 0632, New Zealand; beatrix.jones@gmail.com; 5 Medical Research Council Lifecourse Epidemiology Unit and NIHR Southampton Biomedical Research Centre, University of Southampton and University Hospital Southampton NHS Foundation Trust, Southampton SO16 6YD, UK; kmg@mrc.soton.ac.uk; 6 Department of Maternal Fetal Medicine, KK Women’s and Children’s Hospital, Singapore 229899, Singapore; Kenneth.Kwek.YC@kkh.com.sg; 7 Saw Swee Hock School of Public Health, National University of Singapore and National University Health System, Singapore 117549, Singapore; seang_mei_saw@nuhs.edu.sg; 8 School of Food and Nutrition, Massey University, Auckland 0632, New Zealand; c.conlon@massey.ac.nz; 9 Clinical Nutrition Research Centre, Singapore Institute for Clinical Sciences, A*STAR, Singapore 117609, Singapore

**Keywords:** maternal nutrition, gestational diabetes, Asia, dietary patterns, pregnancy

## Abstract

Gestational Diabetes Mellitus (GDM) is associated with an increased risk of perinatal morbidity and long term health issues for both the mother and offspring. Previous research has demonstrated associations between maternal diet and GDM development, but evidence in Asian populations is limited. The objective of our study was to examine the cross-sectional relationship between maternal dietary patterns during pregnancy and the risk of GDM in a multi-ethnic Asian cohort. Maternal diet was ascertained using 24-h dietary recalls from participants in the Growing up in Singapore towards healthy outcomes (GUSTO) study—a prospective mother-offspring cohort, and GDM was diagnosed according to 1999 World Health Organisation guidelines. Dietary patterns were identified using factor analysis, and multivariate regression analyses performed to assess the association with GDM. Of 909 participants, 17.6% were diagnosed with GDM. Three dietary patterns were identified: a vegetable-fruit-rice-based-diet, a seafood-noodle-based-diet and a pasta-cheese-processed-meat-diet. After adjusting for confounding variables, the seafood-noodle-based-diet was associated with a lower likelihood of GDM (Odds Ratio (95% Confidence Interval)) = 0.74 (0.59, 0.93). The dietary pattern found to be associated with GDM in our study was substantially different to those reported previously in Western populations.

## 1. Introduction

Gestational Diabetes Mellitus (GDM) is a carbohydrate intolerance that develops, or is first recognized during pregnancy [1]. In parallel to the global increase in obesity, the prevalence of GDM has risen substantially [2], affecting certain ethnic groups more than others. Compared with Caucasians, Asians are at a much greater risk of developing GDM at any given Body Mass Index (BMI) [3,4]. Prevalence estimates of GDM in the United States of America ranged from 4.6% to 9.2% [5], whereas estimates in parts of South Asia are as high as 18.9% [6]. 

GDM is associated with both short and long term adverse consequences to expecting mothers and their offspring. Long term consequences to the offspring are thought to arise from modified developmental programming that results from exposure to an aberrant environment within the womb. Adverse consequences of GDM include: an increased risk of delivering an infant with macrosomia; need for a caesarean section; birth trauma, and an increased likelihood of the offspring developing obesity and other metabolic complications in later life. The mother is also at higher risk of developing post-natal type 2 diabetes [7,8,9,10,11,12,13]. 

While a plethora of research has reported associations between maternal nutrient intakes and the development of GDM [14,15,16,17], the single nutrient approach to examining the relationship between diet and disease risk may be complicated due to the interactions among nutrients or the high level of inter-correlation among nutrients [18,19]. Thus, there is growing interest to explore the relationship of maternal diet and GDM using a dietary pattern approach. Dietary pattern analyses have the advantage of accounting for food consumption in a typical diet and take into account the synergy of nutrient intakes as well as their importance in combination. To date, the majority of the studies examining maternal dietary patterns and GDM have been conducted in Western populations [20,21,22]. In general, these studies found that a ‘healthy/prudent’ dietary pattern high in fruit, green leafy vegetables, poultry and fish was associated with a reduced likelihood of GDM, while an ‘unhealthy/Western’ style pattern, high in red meat, processed meat, refined grain products, sweets and desserts, French fries and pizza was associated with an increased likelihood of GDM. Only one other study to our knowledge has investigated dietary patterns in relation to GDM in Asia. From female participants in China, He et al. [23] found that a vegetable-based dietary pattern was associated with reduced rates of GDM, whereas a sweets and seafood based dietary pattern was associated with an increased likelihood of GDM. This finding highlights differences in dietary intakes in an Asian setting and warrants the need to further examine the association between maternal dietary patterns and GDM in Asian women. 

To carry out our study, we investigated maternal dietary patterns using dietary information collected from a large, multi-ethnic Asian cohort established in Singapore.

## 2. Materials and Methods 

### 2.1. Growing up in Singapore towards Healthy Outcomes (GUSTO) Study

The current study was conducted on a sub-set of women from the GUSTO study—a large mother-child cohort that was first established in Singapore in June 2009, with the main aim of evaluating the role of developmental factors in early pathways to metabolic compromise [24]. Participants were recruited in their first trimester of pregnancy from Kandang Kerbau Women’s and Children’s Hospital (KKH) and National University Hospital (NUH). Recruitment was completed in September 2010 (*n* = 1247). Participants were followed throughout pregnancy to delivery, and into the first three years of the offspring’s life, with funding currently in place to continue follow-up until eight years of age. The GUSTO study was approved by the National Health Care Group Domain Specific Review Board (reference D/09/021) and the Sing Health Centralized Institutional Review Board (reference 2009/280/D). Research was conducted according to the Declaration of Helsinki and all participants gave their written informed consent at recruitment. Clinical Trial Registry number: NCT01174875.

GUSTO participants that underwent an Oral Glucose Tolerance Test (OGTT) at 26–28 weeks’ gestation and completed a 24-h dietary recall at the time of OGTT were eligible for our study. Information on maternal ethnicity, age, education level, family history of diabetes, previous history of GDM, and monthly household income was obtained at recruitment, and information on body mass index (BMI), cigarette smoking and alcohol consumption during pregnancy was collected at the time of OGTT. To calculate BMI, maternal weight was measured in duplicate using digital body weight scales (SECA model 803, SECA Corp., Hamburg, Germany) and standing heights were measured with a stadiometer (SECA model 213, SECA Corp.). A multiple imputation procedure [25] was performed using SPSS version 23.0 (SPSS Inc., New York, NY, USA) to impute missing values on the categorical demographic variables of birth order (*n* = 10), education level (*n* = 11), monthly household income (*n* = 58), family history of diabetes (*n* = 33), previous history of GDM (*n* = 33), smoking (*n* = 1) and alcohol consumption (*n* = 21).

### 2.2. Maternal Dietary Assessment

Maternal dietary intake was assessed using a 24-h dietary recall collected at the time of OGTT (26–28 weeks’ gestation). Three-day food diaries were also collected from a small sub-set of participants in the week following OGTT. The dietary recalls were interviewer administered using the five-stage multiple-pass recall interviewing technique [26] in order to optimise the participant’s recall, and all participants were trained by clinical staff on how to correctly complete the three-day food diaries. The dietary information from each participant was coded and entered electronically. Nutrient composition of the foods recorded by participants was assessed using nutrient analysis software (Dietplan, Forestfield Software Limited, Horsham, West Sussex, UK) developed using foods locally available in Singapore. The individual food components in each 24-h recall were assigned into one of 68 food groups that were grouped according to nutrient composition (Online Table S1), and this served as the main dataset for investigation. 

### 2.3. Statistical Analyses

All analyses were performed using SPSS version 23.0 (SPSS Inc.). Exploratory factor analysis with varimax rotation was used for deriving dietary patterns from standardised values of participant food intake from the 68 food groups. Factor analysis is a well-established approach to determine dietary patterns [27,28,29,30]. Orthogonal (varimax) rotation was chosen to allow for the use of dietary pattern scores as predictors in downstream regression analyses. The three dietary patterns that underwent further statistical analyses were selected using two criteria: (1) The number of components identified at the elbow of the scree plot of eigen values following the factor analysis; and (2) The number of components that produced interpretable dietary patterns, relevant to typical food consumption in Singapore. 

Food groups with a loading score of >0.2 were used to describe each dietary pattern (Table S2). Dietary pattern scores for each participant were calculated as the sum of a standardised food group intake multiplied by the corresponding factor loading. 

All foods reported in the three-day food diaries were assigned to one of the 68 pre-defined food groups, as per the 24-h dietary recall analysis. An applied dietary pattern score was calculated for the vegetable-fruit-rice-based-diet and the seafood-noodle-based-diet from each of the 208 participants. Applied scores were calculated by first multiplying the standardised food group intake from the three-day food diaries with the component score of that food group in the dietary pattern derived from the 24-h recalls, then taking the sum of the results from all 68 food groups. Pearson’s Product Moment correlation was performed between the applied scores and the scores from the 24-h recalls.

A binary logistic regression was performed to explore the relationship between the dietary patterns and GDM, and multivariate linear regression was performed to explore the linear association between dietary pattern scores and fasting, and two-hour postprandial glucose levels following OGTT. Both were adjusted for potential confounding variables: maternal age, maternal BMI, energy intake, household income, previous history of GDM, family history of diabetes, ethnicity, education level, birth order, smoking, and alcohol consumption. The potential confounding variables were also analysed for interaction effects with the dietary pattern that was found to be significant in the multivariate model. Identification of interaction effects resulted in the stratification of the data to account for this. Maternal dietary macronutrient composition was compared between participants in the highest and lowest quintile of dietary pattern scores for the dietary pattern of interest. 

For all statistical analyses, a two-tailed *p*-value cut-off of less than 0.05 was considered statistically significant.

## 3. Results

### 3.1. Participant Characteristics

**Figure 1:**
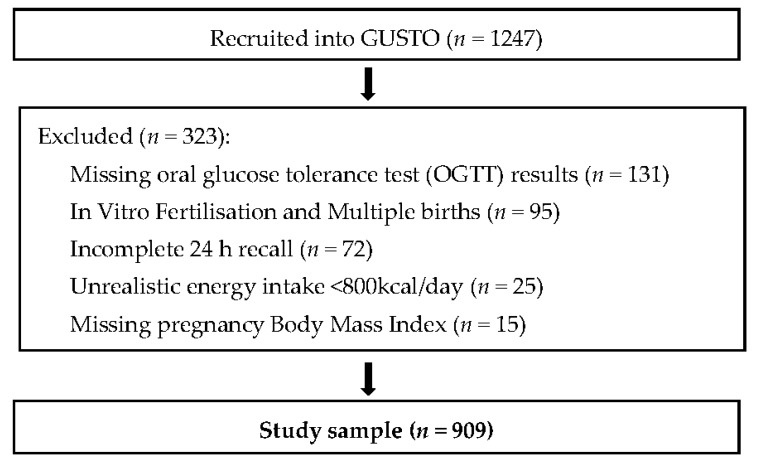
Flowchart showing selection of participants included in this analysis from GUSTO study (Growing up in Singapore towards healthy outcomes), Singapore.

Of the 909 women included in our analyses (Figure 1), 17.6% (*n* = 160) were diagnosed with GDM at the time of OGTT testing (26–28 weeks’ gestation), according to the 1999 World Health Organisation (WHO) diagnostic criteria (WHO, 1999). The mean age of participants was 31 years (±5 years), with a mean BMI of 26 kg/m^2^ (±4.3 kg/m^2^). Participants in our study were predominantly of Chinese ethnicity (56.7%), followed by Malay, (25.7%) and Indian ethnicity (17.6%). Close to half of the participants (*n* = 380, or 41.8%) were nulliparous at recruitment. Women diagnosed with GDM had a significantly higher mean age and BMI when compared with those not diagnosed with GDM. They were also more likely to have completed university education, and reported a higher monthly household income (Table 1). 

### 3.2. Dietary Patterns

Three distinct maternal dietary patterns were identified in our cohort (Table S2). A vegetable-fruit-rice-based-diet high in vegetables, fruit, white rice, bread, low-fat meat and fish, and low in fried potatoes, burgers, carbonated and sugar-sweetened beverages; a seafood-noodle-based-diet high in soup, fish and seafood products, noodles (flavoured and/or in soup), low-fat meat, and seafood, and low in ethnic bread, legumes and pulses, white rice, and curry-based gravies; and a pasta-cheese-processed-meat-diet high in pasta, cheese, processed meats, tomato-based and cream-based gravies. 

#### 3.2.1. Validation of Dietary Patterns

A statistically significant, moderate correlation was observed between participants’ scores on the seafood-noodle-based-diet and vegetable-fruit-rice-based-diet, with applied scores calculated from the three-day food diaries (Vegetable-fruit-rice-based-diet—Pearson’s Product Moment correlation of 0.52, *p* ≤ 0.001; seafood-noodle-based-diet—Pearson’s Product Moment correlation of 0.53, *p* ≤ 0.001).

### 3.3. Dietary Patterns and Gestational Diabetes Mellitus

In unadjusted models, a higher score on the vegetable-fruit-rice-based-diet was associated with a higher likelihood of GDM (Odds Ratio (OR) per Standard Deviation (SD) (95% Confidence Interval (CI)) = 1.37 (1.16, 1.63) *p* < 0.001), while there was a trend towards higher intake of the seafood-noodle-based-diet being associated with a reduction in the likelihood of GDM (OR (95% CI) = 0.85 (0.73, 1.01) *p* = 0.06) (Table 2).

Upon adjustment for confounding variables, the association with the vegetable-fruit-rice-based-diet was no longer significant, with ethnicity being the main effect modifier, however, the interaction effect was not statistically significant (*p* > 0.05). Once stratified by ethnicity, a weak positive association was observed between higher intake of the vegetable-fruit-rice-based-diet and a lower likelihood of GDM, in Chinese participants only (*n* = 505) (OR (95% CI) = 0.75 (0.56, 1.00) *p* = 0.047).

Adjustment for confounding variables strengthened the association between higher scores on the seafood-noodle-based-diet and a lower likelihood of GDM (OR (95% CI) = 0.75 (0.60, 0.93) *p* = 0.008) (Table 2), with a significant interaction effect observed between previous history of GDM and the seafood-noodle-based-diet (*p* for interaction = 0.04). Upon stratification, association of the seafood-noodle-based-diet to GDM only remained statistically significant in women that did not have a previous history of GDM (OR (95% CI) = 0.70 (0.55, 0.88) *p* = 0.002). Compared to participants without a previous history of GDM, those participants with a previous history of GDM had significantly higher consumption of the vegetable-fruit-rice-based-diet (Mean dietary factor scores = −0.014 ± 1.00, 0.40 ± 1.02; *p* = 0.04).

Participants in the highest quintile of seafood-noodle-based-diet consumption were predominantly Chinese, tended to have attained a lower education level, and were less likely to report a family history of diabetes, when compared with women in the lowest quintile of seafood-noodle-based-diet consumption (Table 3). 

### 3.4. Nutrient Composition Comparison between Quintiles of Seafood-Noodle-Based-Diet Consumption

When compared to participants in the lowest quintile, those in the highest quintile of the seafood-noodle-based-diet consumed significantly higher amounts of total energy (1839 vs. 2075 kcal), protein (14.6% vs. 16.3 % of macronutrient intake), total fat (31.2% vs. 33.2 % of macronutrient intake), saturated fat (25 vs. 29 g), and monounsaturated fat (21.41 vs. 27.66 g), and significantly lower amounts of carbohydrate (53% vs. 49.6 % of macronutrient intake) (Table 3). 

### 3.5. Dietary Patterns and Linear Association with Blood Glucose Levels Following OGTT

The vegetable-fruit-rice-based-diet demonstrated a negative linear association with fasting blood glucose levels after adjustment for confounding variables (0.04 mmol/L reduction per SD increase in vegetable-fruit-rice-based-diet score; 95% CI: 0.07, 0.005: *p* = 0.03) (Table 4). Additionally, the vegetable-fruit-rice-based-diet was associated with an increased two-hour blood glucose measurement in an unadjusted model (0.15 mmol/L reduction per SD increase in vegetable-fruit-rice-based-diet score; 95% CI: 0.13, 0.31: *p* ≤ 0.01) (Table 4). However, this relationship weakened and was no longer statistically significant after adjustment for confounding variables. No significant linear association was observed between the seafood-noodle-based-diet and fasting blood glucose. However, there was a marginal negative linear association with 2-h blood glucose levels after adjustment for confounding variables (0.11 mmol/L reduction per SD increase in seafood-noodle-based-diet score; 95% CI: −0.21, 0.001: *p* = 0.05).

## 4. Discussion

We found a significant association between the reported consumption of a seafood-noodle-based-diet during pregnancy, and a lower risk of GDM. Consumption of the vegetable-fruit-rice-based diet was linearly associated with reduced fasting blood glucose, whereas the seafood-noodle-based-diet consumption showed a marginal negative linear association with blood glucose levels two hours following an OGTT 75 g glucose load.

Our study is the first to report findings on the association between maternal dietary patterns and risk of GDM in a multi-ethnic Asian setting. Few studies have investigated the association between maternal dietary patterns during pregnancy and GDM, and to our knowledge only one has done so in an Asian context. He et al. [23] identified four main dietary patterns in their large cohort of Chinese women using a food frequency questionnaire. The highest tertile of vegetable dietary pattern consumption in their cohort was associated with a reduced likelihood of GDM, whereas the highest tertile of the sweets and seafood dietary pattern was associated with an increased likelihood of GDM. Similarly, consumption of the vegetable-fruit-rice-based-diet in our cohort was associated with a lower likelihood of GDM in our Chinese participants, however this association was not found in our Indian or Malay women. Unlike He et al. [23], our vegetable-based dietary pattern (vegetable-fruit-rice-based-diet) also contained white rice. Contrary to our findings, He et al. [23]’s seafood dietary pattern was associated with an increased risk of GDM. This may be explained by the difference in the foods consumed in combination. In our cohort, the seafood was accompanied by soup and noodles, whereas in He et al. [23] the seafood was accompanied by Cantonese desserts and sugar-sweetened beverages.

No studies in the Western population identified rice or noodles in their dietary patterns—two main carbohydrate staples in the Singaporean diet. This may explain the difference between findings in the dietary pattern investigations in Western populations compared to our multi-ethnic Asian cohort. 

Participants in our study that were in the highest quintile of seafood-noodle-based-diet consumption exhibited a higher intake of protein and fat, and a lower intake of carbohydrates in comparison to those in the lowest quintile (Table 3). Previous studies have found an increased risk of GDM with lower carbohydrate intake, particularly when the carbohydrate was substituted for fat [31,32,33]. However, these studies did not take into account the quality of carbohydrate. In the seafood-noodle-based-diet there appears to be a trade-off between white rice and noodle consumption—with those in the highest quintile consuming more noodles and less white rice. Previous research has alluded to a detrimental effect of rice intake in Asian populations, linking higher consumption with an increased risk of Type 2 diabetes [34,35]. White rice has a high glycaemic index [36], particularly when compared with noodles. A high glycaemic index diet during pregnancy has also been associated with GDM [37,38,39]. 

An interaction was observed between previous history of GDM and the seafood-noodle-based-diet. Once stratified by previous history of GDM, the dietary association only remained statistically significant for those who did not have a previous history of GDM. One possible explanation could be that in Singapore women identified as having a previous pregnancy affected by GDM might have received dietary advice to prevent its recurrence in subsequent pregnancies. This dietary advice is most consistent with the vegetable-fruit-rice-based-diet we identified in our cohort. As expected, women with a previous history of GDM had a higher score on the vegetable-fruit-rice-based-diet than those without. 

The use of 24-h dietary recalls to assess typical dietary consumption comes with some limitations. They are subject to recall bias (although this was partly ameliorated by interviewer-administration of recalls), and the mutually exclusive nature of food choice in a 24-h period (dietary variety in some cases may not be assessed to completeness within just 24 h). Validation of the dietary patterns generated from the 24 h dietary recalls was performed using three-day food diaries from a subset of participants (*n* = 208), completed in the week following OGTT. We observed a statistically significant, moderate correlation between participants’ scores on the seafood-noodle-based-diet and vegetable-fruit-rice-based-diet, with applied scores calculated from the three-day food diaries (Vegetable-fruit-rice-based-diet—Pearson’s Product Moment correlation of 0.52, *p* ≤ 0.001; seafood-noodle-based-diet—Pearson’s Product Moment correlation of 0.53, *p* ≤ 0.001). By this criterion, the 24-h dietary recalls adequately represented typical dietary intake of our participants. In addition, we have also shown previously that our 24 h recall has good reproducibility, which provides the basis for the construction of dietary patterns [40]. 

Another limitation of our study is that we cannot rule out the possibility that residual confounding affected the observed associations due to the observational nature of our study [41]. Observational research is important for the early stages of establishing a relationship between diet and disease as they can be used to generate hypotheses that can be tested in validation studies using Randomised Controlled Trials (RCTs).

Odds ratios have some recognised limitations when performed on an outcome that is not rare [42]. As 17.6% of our participants were diagnosed with GDM, the odds ratio value may be subject to inflation. The direction and significance of the relationship remains, but the magnitude of the relationship should be interpreted carefully. To explore this effect, we calculated the relative risk of two participants in our dataset (one considered ‘high-risk’ based on their clinical characteristics and one with ‘low-risk’) with a one standard deviation increase in each of their seafood-noodle-based-diet scores. The relative risk of GDM after one standard deviation increase in the seafood-noodle-based-diet score for our high-risk individual was 0.84, and 0.77 in our low-risk individual.

The strengths of our study include access to the large GUSTO cohort and the dietary pattern approach to investigate the association between maternal diet and GDM. The GUSTO cohort allowed us to address the gap in the literature surrounding maternal dietary patterns and GDM in Asian populations. Dietary pattern investigations take into account foods that are typically consumed in a population and therefore the synergistic impact of nutrients consumed in combination. 

## 5. Conclusions

In summary, our study has demonstrated a significant association between a seafood and noodle-based dietary pattern and the reduced likelihood of GDM in a large, multi-ethnic Asian cohort, based in Singapore. This association is novel, and has not been reported previously. Asian women are at an increased risk of GDM and further research should be conducted in Asian populations to better understand the role of maternal diet in GDM development and progression. This research domain would benefit from studies that can elucidate the metabolic mechanisms linking the protective association of the seafood-noodle-based-diet consumption with GDM.

## Figures and Tables

**Table 1:** Participant characteristics.

Gestational Diabetes Mellitus					
	Yes *n* = 160 (17.6%)		No *n* = 749 (82.4%)		*p*-Value
**Pregnancy BMI** (mean ± SD)	26.6 ± 3.9		25.9 ± 4.4		0.03 *^,a^
**Age** (mean ± SD)	33 ± 5		30 ± 5		<0.001 *^,a^
**Ethnicity** *n* (%)	Chinese	109 (68.1)	Chinese	406 (54.2)	
	Malay	18 (11.2)	Malay	216 (28.8)	
	Indian	33 (20.6)	Indian	127 (17.0)	<0.001 *
**Education** *n* (%)	<Secondary	31 (19.4)	<Secondary	247 (33.0)	
	Post-Secondary	56 (35.0)	Post-Secondary	277 (37.0)	
	University	73 (45.6)	University	225 (30.0)	<0.001 *
**Household Monthly Income** *n* (%)	<2000	14 (8.8)	<2000	113 (15.1)	
	2000–6000	92 (57.5)	2000–6000	447 (59.7)	
	>6000	54 (33.8)	>6000	189 (25.2)	0.02 *
**Smoking** *n* (%)	No	157 (98.1)	No	728 (97.2)	
	Yes	3 (1.9)	Yes	21 (2.8)	0.51
**Alcohol Consumption** *n* (%)	No	156 (97.5)	No	715 (95.5)	
	Yes	4 (2.5)	Yes	34 (4.5)	0.24
**Previous history of GDM** *n* (%)	No	142 (88.8)	No	737 (98.4)	
	Yes	18 (11.2)	Yes	12 (1.6)	<0.001 *
**Family history of Diabetes** *n* (%)	No	104 (65.0)	No	533 (71.2)	
	Yes	56 (35.0)	Yes	216 (28.8)	0.12
**Birth Order** *n* (%)	First child	58 (36.2)	First child	322 (43.0)	
	Not first child	102 (63.7)	Not first child	427 (57.0)	0.12
**Birthweight (g)** (mean ± SD)	3067 ± 494		3098 ± 426		0.47
**Gestational Age at Delivery (days)** (mean ± SD)	269 ± 12		268 ± 33		0.38 ^a^

BMI: body mass index; SD: standard deviation; GDM: gestational diabetes mellitus. Chi-square (2-tailed) tests were conducted to compare group differences, unless otherwise specified. ^a^ Mann-Whitney *U* test was conducted for continuous variables that did not follow a normal distribution. * *p* < 0.05.

**Table 2:** Logistic regression analysis of dietary patterns with GDM outcome.

		Odds Ratio (OR) (95% CI)	*p*-Value
Vegetable-fruit-rice-based-diet			
Unadjusted model		1.37 (1.16, 1.63)	<0.01 *
Multivariate model ^1^		1.10 (0.90, 1.35)	0.36
Seafood-noodle-based-diet			
Unadjusted model		0.85 (0.73, 1.01)	0.06
Multivariate model ^1^		0.74 (0.59, 0.93)	<0.01 *
Pasta-cheese-processed-meat-diet			
Unadjusted model		0.97 (0.81, 1.16)	0.72
Multivariate model ^1^		0.96 (0.79, 1.17)	0.71

CI: confidence interval. ^1^ Adjusted for energy intake, pregnancy BMI, birth order, smoking, alcohol intake, age, ethnicity, education, previous GDM, family history of diabetes, household monthly income, and other dietary patterns. * *p* < 0.05.

**Table 3:** Participant characteristics and nutrient composition by quintile of seafood-noodle-based-diet.

	Quintile 1 of Seafood-Noodle-Based-Diet (Lowest Scores)		Quintile 5 of Seafood-Noodle-Based-Diet (Highest Scores)	*p*-Value
Gestational Diabetes Mellitus *n* (%)	Yes	41 (22.5)	26 (14.3)	0.06 ^b^
	No	141 (77.5)	156 (85.7)	
Pregnancy BMI (mean ± SD)	26.8 ± 4.2		25.4 ± 3.9	0.01 *^,c^
Age (mean ± SD)	30.3 ± 5.3		30.9 ± 4.8	0.34 ^c^
Ethnicity *n* (%)	Chinese	41 (22.5)	154 (84.6)	
	Malay	43 (23.6)	24 (13.2)	
	Indian	98 (53.8)	4 (2.2)	<0.001 *^,b^
Education *n* (%)	<Secondary	41 (22.5)	62 (34.1)	
	Post-Secondary	60 (33)	64 (35.2)	
	University	81 (44.5)	56 (30.8)	0.01 *^,b^
Household Monthly Income *n* (%)	<2000	21 (11.5)	22 (12.1)	
	2000–6000	108 (59.3)	98 (53.8)	
	>6000	53 (29.1)	62 (34.1)	0.55 ^b^
Smoking *n* (%)	No	173 (95.1)	179 (98.4)	0.14 ^b^
	Yes	9 (4.9)	3 (1.6)	
Alcohol Consumption *n* (%)	No	175 (96.2)	174 (95.6)	0.99 ^b^
	Yes	7 (3.8)	8 (4.4)	
Previous history of GDM *n* (%)	No	178 (97.8)	172 (94.5)	0.17 ^b^
	Yes	4 (2.2)	10 (5.5)	
Family history of Diabetes *n* (%)	No	110 (60.4)	140 (76.9)	0.001 *^,b^
	Yes	72 (39.6)	42 (23.1)	
Birth Order *n* (%)	First Child	65 (35.7)	80 (44.0)	
	Not first child	117 (64.3)	102 (56.0)	0.13 ^b^
Birthweight (g) (mean ± SD)	3070 ± 388		3141 ± 456	0.12 ^c^
Gestational Age at Delivery (days) (mean ± SD)	269 ± 30		268 ± 30	0.87 ^c^
Energy (kcal)	1839 (1474, 2301) ^a^		2075 (1735, 2445) ^a^	<0.001 *^,d^
Nutrient Composition				
Protein, % of Energy	14.6 (12.5, 17.3) ^a^		16.3 (13.7, 18.6) ^a^	<0.001 *^,d^
Total Fat, % of Energy	31.2 (25.0, 37.7) ^a^		33.2 (29.0, 38.1) ^a^	0.004 *^,d^
Carbohydrate, % of Energy	53.0 (47.5, 60.9) ^a^		49.6 (44.2, 55.4) ^a^	<0.001 *^,d^
Saturated Fat (g)	25 (15, 34) ^a^		29 (22, 40) ^a^	<0.001 *^,d^
Monounsaturated Fat (g)	21.41 (14.77, 29.60) ^a^		27.66 (21.03, 41.01) ^a^	<0.001 *^,d^
Polyunsaturated Fat (g)	10.9 (6.9, 16.5) ^a^		11.8 (8.1, 16.2) ^a^	0.13 ^d^
Protein (g)	69.13 (51.17, 85.33) ^a^		81.38 (67.96, 101.71) ^a^	<0.001 *^,d^
Carbohydrate (g)	244.48 (198.09, 303.00) ^a^		254.98 (204.18, 305.66) ^a^	0.27 ^d^
Total Fat (g)	64.71 (43.52, 85.83) ^a^		75.38 (59.74, 100.69) ^a^	<0.001 *^,d^

^a^ Median (25th percentile, 75th percentile); ^b^ Chi-square (2-tailed) tests; ^c^ Independent samples *t*-test; ^d^ Mann-Whitney *U* test; * *p* < 0.05.

**Table 4:** Linear regression analysis of dietary patterns with fasting and 2-hour blood glucose levels following OGTT.

	Fasting Blood Glucose β (95% CI)	*p*-Value	2-Hour Blood Glucose β (95% CI)	*p*-Value
Vegetable-fruit-rice-based-diet				
unadjusted model	−0.03 (−0.04, 0.02)	0.36	0.15 (0.13, 0.31)	<0.01 **
Multivariate model ^1^	−0.04 (−0.07, –0.005)	0.03 *	0.08 (−0.02, 0.18)	0.11
Seafood-noodle-based-diet				
Unadjusted model	−0.05 (−0.05, 0.007)	0.14	−0.04 (−0.15, 0.04)	0.27
Multivariate model ^1^	−0.01 (−0.05, 0.02)	0.44	−0.11 (−0.21, 0.001)	0.05
Pasta-cheese-processed-meat-diet				
Unadjusted model	−0.05 (−0.05, 0.008)	0.16	−0.05 (−0.17, 0.02)	0.13
Multivariate model ^1^	−0.006 (−0.03, 0.02)	0.70	−0.07 (−0.16, 0.03)	0.16

^1^ Adjusted for energy intake, pregnancy BMI, birth order, smoking, alcohol consumption, age, ethnicity, education, previous GDM, family history of diabetes, and household monthly income. * *p* < 0.05, ** *p* < 0.01.

## Supplementary Materials

The following are available online at http://www.mdpi.com/2072-6643/8/9/574/s1, Table S1: List of the 68 Food Groups, and Table S2: Three dietary patterns identified from Exploratory Factor Analysis using Varimax Rotation.
