# Supplementary material for: Maternal Dietary Patterns and Gestational Diabetes Mellitus in a Multi-Ethnic Asian Cohort: The GUSTO Study
Source: Nutrients. 2016 Sep 20;8(9):574. doi: 10.3390/nu8090574 (PMC5037559; doi:10.3390/nu8090574)
Supplement: Supplementary file 1 [file nutrients-08-00574-s001.docx]

Supplementary Materials: Maternal Dietary Patterns and Gestational Diabetes Mellitus in a Multi-Ethnic Asian Cohort: The GUSTO Study

Jamie de Seymour, Airu Chia, Marjorelee Colega, Beatrix Jones, Elizabeth McKenzie, Cai Shirong, Keith Godfrey, Kenneth Kwek, Seang-Mei Saw, Cathryn Conlon, Yap-Seng Chong, Philip Baker and Mary F. F. Chong

**Table S1.** List of the 68 food groups.

| **Food Groups** | | | |
| --- | --- | --- | --- |
| Poultry (B) | Grains | Cheese |  |
| Red Meat (B) | Oats | Other dairy |  |
| Red Meat (F) | Breakfast Cereals/Bars | Carbonated Drinks |  |
| Meat Products | Butter/Ghee | Sweetened Drinks |  |
| Meat Innards | Margarine/Peanut Butter | Soya Bean Drinks |  |
| Fish (B) | Salad Dressing | Coffee/Tea |  |
| Fish (F) | Cream-based Soup | Dessert Soup |  |
| Seafood | Soya Sauce-based Gravy | Local Sweet Snacks/Pastries/Biscuits |  |
| Fish/Seafood Products | Other Gravy | Local Savoury Snacks (B) |  |
| Eggs | Sweet Condiments | Local Savoury Snacks (F) |  |
| Vegetables | Chocolate | Ice cream |  |
| Starchy Vegetables | Noodles in Soup | Chips |  |
| Potatoes (F) | Flavoured Noodles | Blended Oil |  |
| Fresh Fruit | Pasta | MUFA/PUFA Oil |  |
| Fruit Juice | White Bread | Meat/Vegetable Soup |  |
| Processed Fruit | Wholemeal/Multigrain Bread | Tomato-based Gravy |  |
| Beancurd | Ethnic Bread | Cream-based Gravy |  |
| Legumes/Pulses | Bread with Toppings | Curry-based Gravy |  |
| Nuts/Seeds | Low-Fat Milk | Sweets |  |
| White Rice | Whole Milk | Sweet spreads |  |
| Brown Rice | Formula Milk | Burger |  |
| Flavoured Rice | Milk-Based Drinks | Pizza |  |
| Porridge | Yogurt & Cultured Drinks |  |  |

F = Fried preparation or curry cooked in coconut; B = Boiled, steamed, grilled, roasted, baked, stir fried, braised, or stewed preparation.

**Table S2.** Three dietary patterns identified from exploratory factor analysis using varimax rotation.

| **Vegetable-Fruit-Rice-Based-Diet** | | **Seafood-Noodle-Sased-Diet** | | **Pasta-Cheese-Processed-Meat-Diet** | |
| --- | --- | --- | --- | --- | --- |
| Food Group | Factor Loading Coefficient | Food Group | Factor Loading Coefficient | Food Group | Factor Loading  Coefficient |
| Cruciferous, leafy, yellow and orange vegetables | 0.54 | Soup | 0.54 | Pasta | 0.81 |
| Other vegetables | 0.45 | Fish and Seafood Products | 0.43 | Tomato-based Gravies | 0.70 |
| Fruits | 0.40 | Noodles (in Soup) | 0.41 | Cheese | 0.46 |
| White Rice | 0.36 | Flavoured Noodles | 0.39 | Cream-based Gravies | 0.46 |
| Soup | 0.32 | Non-fried red meat | 0.35 | Processed meats | 0.26 |
| Wholegrain bread | 0.29 | Seafood | 0.29 |  |  |
| Ethnic Bread | 0.23 | Soya sauce based Gravies | 0.26 |  |  |
| Non-fried Fish | 0.22 | Processed meats | 0.20 |  |  |
| Legumes and Pulses | 0.21 | Local Savoury Snacks (not fried) | 0.20 |  |  |
| Nuts & Seeds | 0.21 | Other Grains | –0.22 |  |  |
| Milk-based drinks | –0.22 | Curry-based Gravies | –0.28 |  |  |
| Red Meat (Deep fried/in curry) | –0.25 | White Rice | –0.28 |  |  |
| Sugar-sweetened beverages | –0.26 | Legumes and Pulses | –0.40 |  |  |
| Flavoured Rice | –0.30 | Ethnic Bread | –0.43 |  |  |
| Burgers | –0.32 |  |  |  | |
| Carbonated drinks | –0.33 |  |  |  | |
| Fried Potatoes | –0.41 |  |  |  | |

Extraction method: principal component analysis; rotation method: varimax with kaiser normalization; loading factors < 0.20 were not listed in the table for simplicity.
